# Supplementary material for: Objective monitoring of functional recovery after total knee and hip arthroplasty using sensor-derived gait measures
Source: PeerJ. 2022 Sep 28;10:e14054. doi: 10.7717/peerj.14054 (PMC9526408; doi:10.7717/peerj.14054)
Supplement: Supplemental Information 1 — Supplementary 1 - types of hip implants Suplplementary 2 - missing data and complications [file peerj-10-14054-s001.docx]

**Supplementary 1 – types of hip implants**

Fourteen patients with hip OA received an uncemented acetabular component (Allofit Alloclassic®) with Cementless Spotorno (CLS) stem (Zimmer Biomet, Warsaw, IN). Seven patients received the same acetabular component, but with a cemented Müller stem (Zimmer Biomet, Warsaw, IN) (n=6) or uncemented Wagner Cone Stem (Zimmer Biomet, Warsaw, IN) (n=1). Finally, three patients received a cemented Müller cup (Smith & Nephew, Memphis, TN) with cemented Müller stem (Zimmer Biomet, Warsaw, IN). In all patients a ceramic femoral head (Biolox ®) and polyethylene insert (Durasul ®) was used.

**Supplementary 2 – Missing data and complications**

Two participants (1 TKA, 1 THA) were unable to complete the gait test without assistive device at two months after surgery. Fifteen months after surgery, three (THA) participants did not participate due to COVID-19 related reasons, two (THA) received a contralateral arthroplasty, one (TKA) moved abroad, one (TKA) was unable to complete the gait test without assistive device, one (THA) did not want to participate for unspecified reasons, and one study visit (TKA) fell outside the study window. Regarding post-operative complications, one patient had stiffness after TKA with good recovery after manipulation under anesthesia (three months post-operatively), one patient had an avulsion fracture of the trochanter major 8 days after THA (without readmission), and one patient had a revision for dislocation after THA (five months post-operatively) with no follow-up measurement at 15 months for COVID-19 related reasons.
